# Computational Analysis of Machining Induced Stress Distribution during Dry and Cryogenic Orthogonal Cutting of 7075 Aluminium Closed Cell Syntactic Foams

**DOI:** 10.3390/mi14010174

**Published:** 2023-01-10

**Authors:** Kevin K. Thomas, Sathish Kannan, Salman Pervaiz, Mohammad Nazzal, Ramanujam Karthikeyan

**Affiliations:** 1 Department of Mechanical Engineering, American University of Sharjah, Sharjah P.O. Box 26666, United Arab Emirates; 2 Department of Mechanical Engineering, Rochester Institute of Technology, Dubai P.O. Box 341055, United Arab Emirates; 3 Department of Mechanical Engineering, BITS Pilani, Dubai P.O. Box 345055, United Arab Emirates

**Keywords:** machining, aluminum, syntactic foam, surface integrity, FE model

## Abstract

The addition of hollow aluminium oxide bubbles to the 7075 aluminium matrix results in a lightweight syntactic foam with a reduced density and an increased peak compression strength. The presence of ceramic bubbles also aids in a reduced coefficient of thermal expansion and thermal conductivity in comparison to aluminium alloys. In spite of their enhanced material properties, the inclusion of hollow aluminium oxide bubbles presents the challenge of poor machinability. In order to elucidate the problem of poor surface machinability, an attempt has been made to develop a thermo-mechanical finite element machining model using AdvantEdge^TM^ software with which surface quality and machined syntactic foam material can be analyzed. If the novel model developed is combined with virtual reality technology, CNC technicians can observe the machining results to evaluate and optimize the machining program. The main novelty behind this software is that the material foam is assumed as a homogeneous material model for simplifying the material model as a complex heterogeneous material system. The input parameters used in this study are cutting speed, feed, average size and volume fraction of hollow aluminium oxide bubbles, and coolant. For the output parameters, the numerical analysis showed a 6.24% increase in peak tensile machining induced stress as well as a 51.49% increase in peak cutting temperature as cutting speed (25 m/min to 100 m/min) and uncut chip thickness (0.07 mm to 0.2 mm) were increased. The average size and volume fraction of hollow aluminium oxide bubbles showed a significant impact on the magnitude of cutting forces and the depth of tensile induced stress distribution. It was observed on the machined surface that, as the average size of hollow aluminium oxide bubbles became coarser, the peak machining induced tensile stress on the cut surface reduced by 4.47%. It was also noted that an increase in the volume fraction of hollow aluminium oxide bubbles led to an increase in both the peak machining induced tensile stress and the peak cutting temperature by 29.36% and 20.11%, respectively. This study also showed the influence of the ceramic hollow bubbles on plastic deformation behavior in 7075 aluminium matrix; the machining conditions for obtaining a favorable stress distribution in the machined surface and sub-surface of 7075 closed cell syntactic foam are also presented.

## 1. Introduction

Aluminum syntactic foams are manufactured by the insertion of abrasive micro/nano-sized hollow bubbles into the matrix alloy. This class of lightweight material finds potential applications in the automotive sector for the manufacture of front suspension casting, bumper housing casting, and side-impact beams [1]. This unique kind of foam, also known as closed-cell foam, holds outstanding characteristics, such as a high strength to weight ratio, a reduced thermal expansion coefficient, high thermal conductivity and good damage tolerance, making it a potential candidate for applications involving energy absorption [2,3]. Moreover, it can be used as backup plates used in armor ballistic plates and bulletproof vests as the syntactic foam has the benefits of a higher peak strength and an exceptional energy absorbing capability [4,5]. As the syntactic foam has similar characteristics to aluminium (Al) foams, it can be useful to the production of automotive brake rotors as well as of differential covers. Aluminium syntactic foam may be useful in brake lining applications owing to its better wear resistance [6,7]. Every automotive industry has to prioritize tackling the problem of weight reduction and fuel efficiency by decreasing environmental pollution and emissions of hazardous gases. Based on this, there is a good chance that the syntactic foams will be applicable in the bumper beam and pillar reinforcements of a car, which would result in the achievement of weight reduction [8]. Owing to their lightness and ability to absorb high energy, aluminium syntactic foams can be used to improve the armor characteristics of military vehicles and blast-resistant structures [9].

These characteristics have encouraged researchers to conduct force analyses during the machining of aluminium syntactic foams, which could help to synthesize advanced useful products at a reduced production cost. Bolat et al. [10] conducted face turning on pumice reinforced aluminium alloy 7075 syntactic foams to investigate the foams’ machining characteristics. The authors notably deduced that cutting forces (cutting, feed, and radial forces) are higher when the cutting speed is increased at constant feed rate owing to the matrix softening and the porosity present in pumice particles. Kannan et al. [11] explained the machining mechanics of 7075 syntactic foam with the help of an analytical force model. The key conclusions put forth by the authors were (a) that the plastic deformation of 7075 matrix is influenced by the pinning action of hollow aluminium oxide bubbles present inside the matrix, and (b) that the predicted cutting forces are higher with increased volume fraction and a coarser average size of bubble. Kannan et al. [12] also analyzed the machining characteristics of AZ91 magnesium alloy syntactic foam. An important observation by the authors is the increase in cutting force values by 100N due to the aluminium oxide bubble pinning the matrix along the grain boundaries, which will lead to higher strain hardening in the matrix. Alhourani et al. [13] created an analytical model for explaining the machinability of AZ31 magnesium syntactic foam reinforced with hollow aluminium oxide bubbles. A vital conclusion from the author’s results is the sudden increase in friction forces with an increasing volume fraction of hollow aluminium oxide bubbles. Thomas et al. [14] observed an increase in cutting forces with higher uncut chip thickness during the development of a two-dimensional (2D) finite element (FE) model for studying the machinability of 6061 matrix reinforced with Al_2_O_3_ bubbles during orthogonal machining. Ullen [15] evaluated the machinability of Cu-Ni-Mo-based steel foams with varying porosities produced by powder metallurgy. One of his findings was a reduction in thrust forces when porosity content increased, mainly due to the brittle fractures around the pores leading to reduced cutting forces. Guerra-Silva et al. [16] performed an analysis on the influence of machining conditions and tool geometrical features on the orthogonal machining of cellular heat-resistant austenitic stainless steel by means of an FE simulation model. The key conclusion drawn by the authors is the increased cutting force with higher cutting speed and/or feed rate due to the non-homogeneous nature of the metal (presence of foam). From the papers mentioned, very few authors developed a force model for the force analysis of syntactic foams.

Although several studies on force analysis have been mentioned, the main aim of this paper is to study the surface quality and integrity of 7075 based syntactic foams. As it is a material which has been recently developed, there are only limited studies related to 7075 based syntactic foams. Since the syntactic foams belong to the family of metal matrix composites (MMC), there are several surface integrity studies analyzing MMC. One of the studies conducted by Zou et al. [17] was on the effect of machining parameters on the residual stress distribution during orthogonal machining of aluminum matrix reinforced with SiC particles. The authors were able to observe the residual stress on the machined surface become more tensile in nature with the increase in cutting speed. A similar trend was observed for higher uncut chip thickness. Xiang et al. [18] conducted an experimental study on the surface integrity during high-speed milling of Al6063 matrix composites. Two main points discussed by the authors were that (a) surface compressive residual stresses decreased by 81 MPa as the feed rate increased from 0.02 to 0.1 mm/rev as a larger removal rate raises the temperature to induce tensile stress, and that (b) surface tensile residual stress increased with an increase in cutting speed due to the high heat generated during milling at higher cutting speeds. The cutting speed effect was similarly observed by Xiong et al. [19]. Lin et al. [20] analyzed the effect of tool nose radius and tool wear on the residual stress distribution during turning of 7075 matrix reinforced with TiB_2_ particles. The authors found that the surface residual stress is more tensile in nature with the increase in tool nose radius, and that tool wear has more influence on the machining induced stress distribution than the nose radius. Claub et al.’s [21] investigation was into the effect of cutting parameters and clearance angle on the surface properties during turn milling of aluminium matrix composites using monocrystalline diamond tool. Two notable observations were made by the authors: (a) at higher clearance angle, a negligible change in compressive residual stress was observed when the cutting speed was higher; and (b) at lower clearance angle, reduction in compressive residual stress was observed when the cutting speed was higher. In all the surface integrity studies mentioned, residual stress distribution was initially compressive in nature, and, after a certain depth beneath the machined surface, the distribution was tensile for MMC. This trend was observed even through simulation analysis by Li et al. [22] using ABAQUS/Explicit. From these studies no information was given about the machining induced stress distribution for syntactic foams using FE analysis.

Although machinability analysis and surface integrity analysis have been performed on metal syntactic foams and metal foams, there are few studies to explain the interaction between hollow bubble reinforcement and matrix using the FE method. In this paper, an effort has been made to develop a 2D FE model using AdvantEdge^TM^ software for the prediction of cutting forces and distribution of machining peak cutting temperature during the machining of 7075 aluminium syntactic foams. Within the FE model, machining induced stress distribution below the machining layer is forecasted and analyzed. The stress distribution analysis will enable the identification of favorable machining conditions and average size and volume fraction of hollow aluminium oxide bubbles, which will help in the improvement of products’ performance during service.

This aim of this paper is the development of a simplified FE model which can be used by production engineers to characterize cutting forces and peak cutting temperature distribution when machining closed-cell syntactic foams. This simplification in the model will aid in visualizing the machining induced stresses, which is vital for understanding the fatigue life of components in service. This FE model aims for a reduction in the time and production costs required for modelling complex syntactic foam materials, via visualizing machining induced quality defects on different components. To the author’s knowledge, this machining model is an entirely new model developed for closed-cell syntactic foams. Machining tests were conducted for the verification of the developed FE model, which showed reasonable agreement with the experimental values. The novelty behind this software is that the material foam is assumed as a homogeneous material model in order to simplify the material model as a complex heterogeneous material system for FE simulations. With the two-dimensional FE force model developed, it is possible to generate machining induced stress distribution graphs from the contours for syntactic foams.

## 2. Materials and Methods

### 2.1. Numerical Model of Machining Aluminum Closed Cell Syntactic Foams

**Figure 1:**
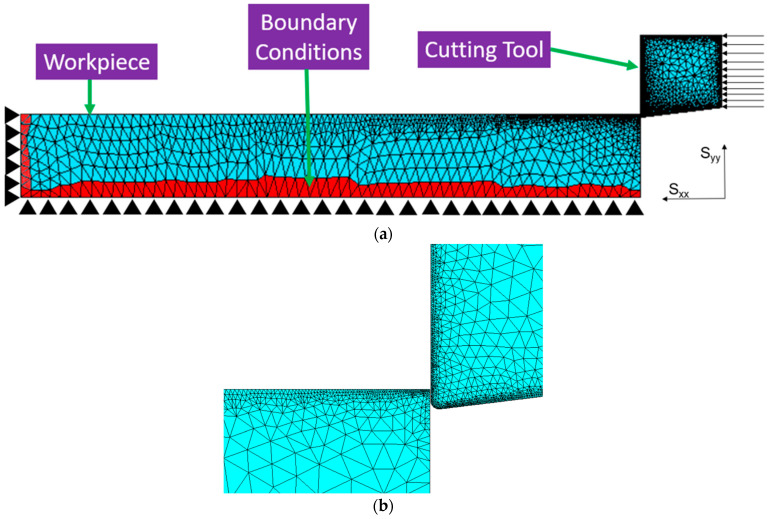
(**a**) Boundary conditions and meshing employed for workpiece and machining tool used in numerical prediction model for AdvantEdge^TM.^; (**b**) Zoomed in version of tool and workpiece.

For simulating cutting forces, peak cutting temperature distribution, and machining induced stresses generated in the process, AdvantEdge^TM^ 7.7, a numerical prediction software developed by Third Wave Systems USA, is implemented. The machining model developed in combination with virtual reality technology creates a potential opportunity for CNC technicians to observe the machining results for optimizing the machining program before actual machining [23]. For modelling the workpiece in the AdvantEdge^TM^ software, the maximum workpiece length used is 15 mm depending on the required accuracy. For boundary conditions, constraints are applied on the left side and base side of the workpiece such that both sides are prohibited from moving in S_xx_ and S_yy_ directions, as shown in Figure 1. This is done so that the workpiece will remain fixed, and the tool moves only in S_xx_ direction during chip removal. Therefore, a relative movement between the machining tool and the syntactic foam (workpiece), also known as cutting speed, will be developed. The boundary conditions for the cutting speed and feed of the machining tool are established with respect to the reference point, which is located at the cutting edge of the tool. For meshing, tri-element mesh is used for both the machining tool and the workpiece as it is the only element offered by the AdvantEdge^TM^ software. For the reduction of computational time, the minimum and maximum mesh sizes of the workpiece used in the AdvantEdge^TM^ software are 20 μm and 100 μm, respectively. The software allows the input of the number of nodes for the workpiece, specifically 72,000, instead of the number of elements. Depending on the cutting speed, feed rate, and average size and volume fraction of hollow aluminium oxide bubbles used in this study, loads in the range of 306–799 N will be uniformly distributed on the cutting tool in S_xx_ direction. The force values will be discussed in the results. 

### 2.2. Material Model 

In AdvantEdge^TM^, the correlation between flow stress and peak equivalent plastic strain is elucidated by a user-defined yield surface material model, which is related to Johnson and Cook (JC) model constants. In this software, the material foam model is presumed to be a homogenous model in order to simplify the material model as a complex heterogeneous material system. The chemical composition of 7075 syntactic foam is detailed in Table 1, and the physical and mechanical properties of 7075 matrix and hollow aluminium oxide bubbles are shown in Table 2. 

**Figure 2:**
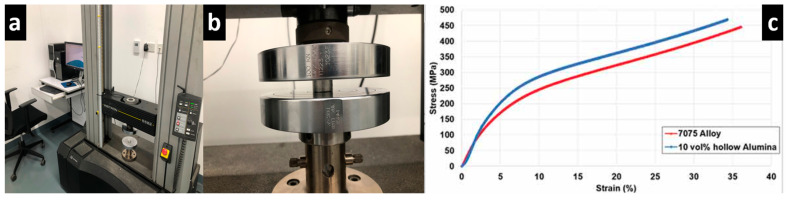
(**a**) Different views of compression test set-up; (**b**) a close-up view of 7075 Aluminum sample kept between pressure plates during compression; (**c**) Stress-strain compression behavior of 7075 Aluminium.

The response for plastic deformation by 7075 matrix is modelled using the JC material model. The JC model depends on the flow stress on the strain, strain rate, and peak cutting temperature. The constants for the JC model are calculated from tests involving stress, strain, and peak cutting temperature. A set of compression tests is conducted on the squeeze casted samples at different strain rates and peak cutting temperatures using the 3400 series of the Instron universal testing machine, which is capable up to 100 kN (as shown in Figure 2a,b), through which material constants are calculated. A stress–strain datum which represents 7075 syntactic foam is shown in Figure 2c. On the basis of mechanical testing conducted at different peak cutting temperatures and strain rates, a quasi-static compression test is used to calculate yield stress (A = 170 MPa) and strain hardening variables (B = 658 MPa and n = 0.53) at reference strain rate (1/s) and peak cutting temperature (20 °C) [11]. The mechanical tests which are conducted at different strain rates at fixed peak cutting temperature are used for determining the dimensionless strain rate sensitivity parameter (C = 0.019). The tests performed for different peak cutting temperatures at a uniform strain rate are also used for calculating the thermal softening coefficient (m = 0.32). The main characteristics of 7075 matrix and alumina bubble (supplier data) are mentioned in Table 2, and the properties of the machining tool material, which are used in AdvantEdge^TM^, are mentioned in Table 3.

### 2.3. Chip Separation Criterion

In general, for any FE machining simulation software including AdvantEdge^TM^, the most important part of any orthogonal machining simulation is the criteria for chip separation. In normal situations, there are two methods for chip separation: node-splitting and element deletion methods. For a node splitting case, a chip separation plane is predefined, and a separation criterion is applied. There are two types of separation criteria: geometrical and physical. Because the geometrical criteria do not have physical meanings, they are considered a weaker separation technique in comparison to physical ones, making physical ones more preferable for chip separation. Physical separation criteria are developed on the basis of the critical value of a physical quantity for estimation of the onset of chip separation. Based on the user value of reference strain rate present in the JC flow stress equation, a critical peak equivalent plastic strain to chip separation is employed. The damage variable evolution is assumed to have a linear relationship with effective plastic displacement. The software provides an option to enter the effective plastic displacement, ${\bar{A}}_{F}^{PL}$, at the point of failure. The damage variable *D* is increasing based on the equation [28]:

(1) $$D=\frac{{\dot{\bar{A}}}^{PL}}{{\bar{A}}_{F}^{PL}}$$

The chip separation criteria are enabled through the material failure when the ratio of variable plastic displacement to effective plastic displacement becomes 1. 

### 2.4. Chip–Tool Interaction

In AdvantEdge^TM^, the sliding friction force is linearly proportional to the applied normal load. The constant of proportionality for this equation is the coefficient of friction, which will not vary in all secondary shear zones between the deformed chip and cutting tool for different machining parameters. The frictional conditions between tool and chip in AdvantEdge^TM^ are modelled by the Coulomb friction model which is shown below [28].

(2) $$F_{FR}=\mu n$$

where $F_{FR}$ is the friction force exerted between the surfaces, *µ* is the coefficient of friction, and *n* is the normal force.

### 2.5. Experimental Validation

**Figure 3:**
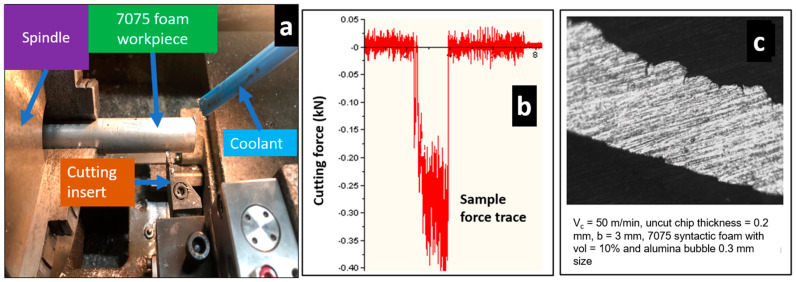
(**a**) Machining experimental test setup; (**b**) Sample force graph taken from dynamometer; (**c**) Representative machined chip for 7075 syntactic foam.

To corroborate the simulated force values from the 2D FE model for 7075 syntactic foams, machining experiments are performed on 7075 reinforced with hollow aluminium oxide bubbles (see Figure 3a). The complete information regarding the machining parameters is shown in Table 4. The selection of parameters was based on the paper selected from Kannan et al. [11]. The machining tool used in the tests is a Kennametal coated carbide insert, which has a 0° rake angle and 7° clearance angle. A KISTLER™ dynamometer with a multichannel charge amplifier is used in measuring cutting forces from a force trace graph. The forces are measured as soon as the cutting tool makes contact with the workpiece during machining, and the forces are taken by averaging the constant values of forces in the force trace graph shown in Figure 3b. Machining tests are carried out twice to guarantee repeatability, and average measurement values are noted down. Due to constraints on 7075 foam test piece availability, the number of experiments had to be limited by the authors. In order to perform cryogenic machining, a liquid nitrogen coolant with a thermal conductivity of 61.610 kW m^−2^K^−1^ and coolant peak cutting temperature of −185.22 °C [29] was used. The liquid nitrogen was delivered to the entire workpiece sample by means of a hose connected to a liquid nitrogen tank. The experiments were carried out to measure cutting force values which are already presented in Kannan et al. [11]. These force values are compared to FE cutting forces whose magnitudes are presented in the results. The experiments for measuring peak equivalent plastic strain, peak cutting temperature, and machining induced stresses were not conducted, but their values generated from the 2D FE model are also shown in the results.

## 3. Results and Discussion

### 3.1. Cutting Forces and Peak Cutting Temperature Distribution

**Figure 4:**
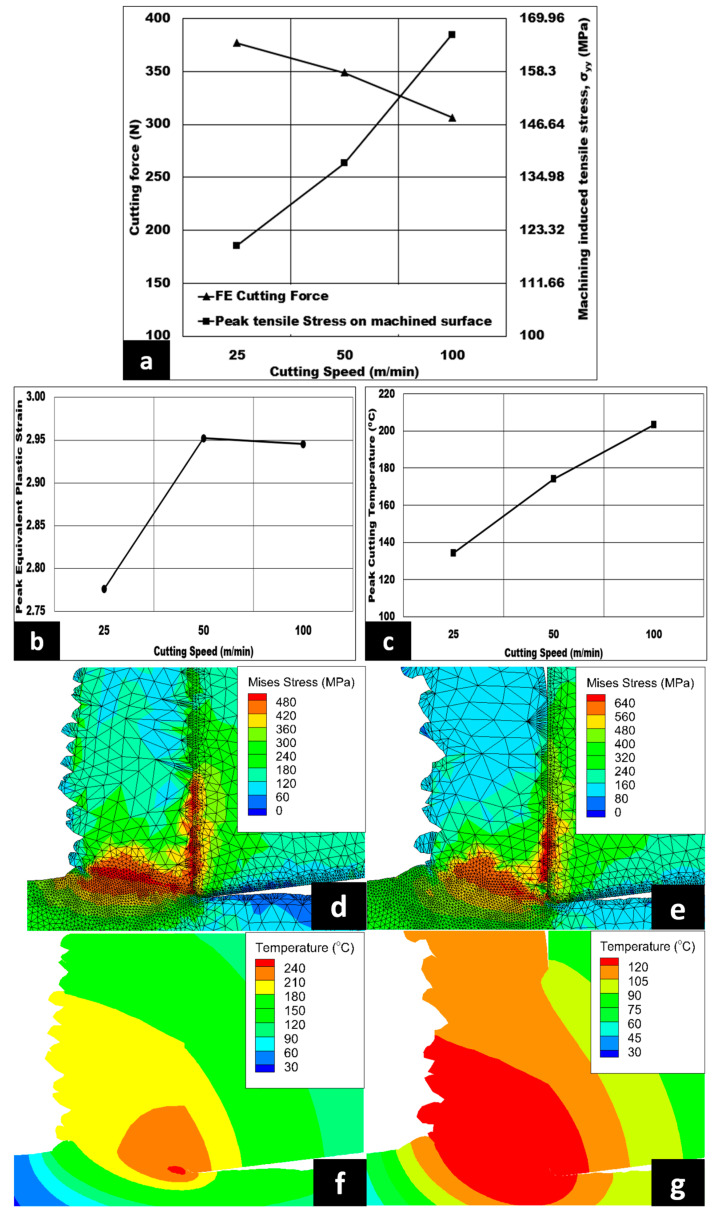
(**a**–**c**) Variation of cutting velocity on cutting forces, machining induced stress on machined surface, peak equivalent plastic strain, and peak cutting temperature (AdvantEdge^TM^ simulation) under dry conditions. 7075/hollow alumina shell syntactic foams (vol% = 10%, h = 0.07 mm, b = 3 mm, Dry cut); Von Mises stress contour at (**d**) 100 m/min and (**e**) 25 m/min; peak cutting temperature contour at (**f**) 100 m/min and (**g**) 25 m/min.

Figure 4a–c reveals the influence of cutting speed on the magnitude of generated cutting force, cutting induced peak tensile stress (measured on the machined surface), peak equivalent plastic strain, and peak cutting temperature under dry cutting. The cutting force decreases in a linear manner from 377 N to 306 N as the cutting speed is increased from 25 m/min to 100 m/min. From the numerical analysis, the machining induced peak tensile stress increases by 38.74% when the cutting speed is increased. The peak equivalent plastic strain in the shear zone increases from 2.78 to 2.95, which is approximately about 6.1%, when the cutting speed is increased. Although the strain is expected to increase, the negligible drop in plastic strain from 50 m/min to 100 m/min could be due to the assumption made in the model. Being one of the few models developed for aluminium based syntactic foams, the plastic strain (also known as equivalent plastic strain) contour values were comparable to Josyula and Narala’s paper, which discusses TiC particle distribution in aluminium based composite using FE [30]. Variation in peak cutting temperature is also observed, with an increase of 50% as the cutting speed is increased from 25 m/min to 100 m/min. Figure 4d,e shows the influence of cutting speed on the variation of Von Mises stress contours. As seen from the contour, a maximum value of 640 MPa is noted at 25 m/min. From Figure 4f,g, the influence of cutting speed on peak cutting temperature contours is shown, with a maximum value of 240 °C at 100 m/min.

When the cutting speed is raised, the length of the shear plane is increased, causing the shear angle to drop. Therefore, the shear plane area becomes reduced, causing the shear force required and subsequently the shear stress to decrease, which in turn causes the cutting force to be lowered (refer to Figure 4a). This is also evident from the change in Von Mises stress contour shown in Figure 4d,e as the cutting speed drops from 100 m/min to 25 m/min. In Figure 4c, the peak cutting temperature value is found to be increased with the increase in cutting speed, owing to the thermal softening of 7075 matrix. This phenomenon happens when the strain rate is increased from 5952.39 s^−1^ to 23,809.6 s^−1^ as the cutting speed increases. Subsequently, this will lead to an increased coefficient of friction between the machining tool and the syntactic foam. Therefore, heat dissipation is more concentrated on the shear zone and softens the 7075 matrix. Consequently, higher cutting speed results in increased strain rate, peak equivalent plastic strain, and higher cutting temperature, so the machining induced stress is found to be more tensile in nature, as observed in Figure 4a. These trends are found to be in agreement with those observed by Thomas et al. [14].

**Figure 5:**
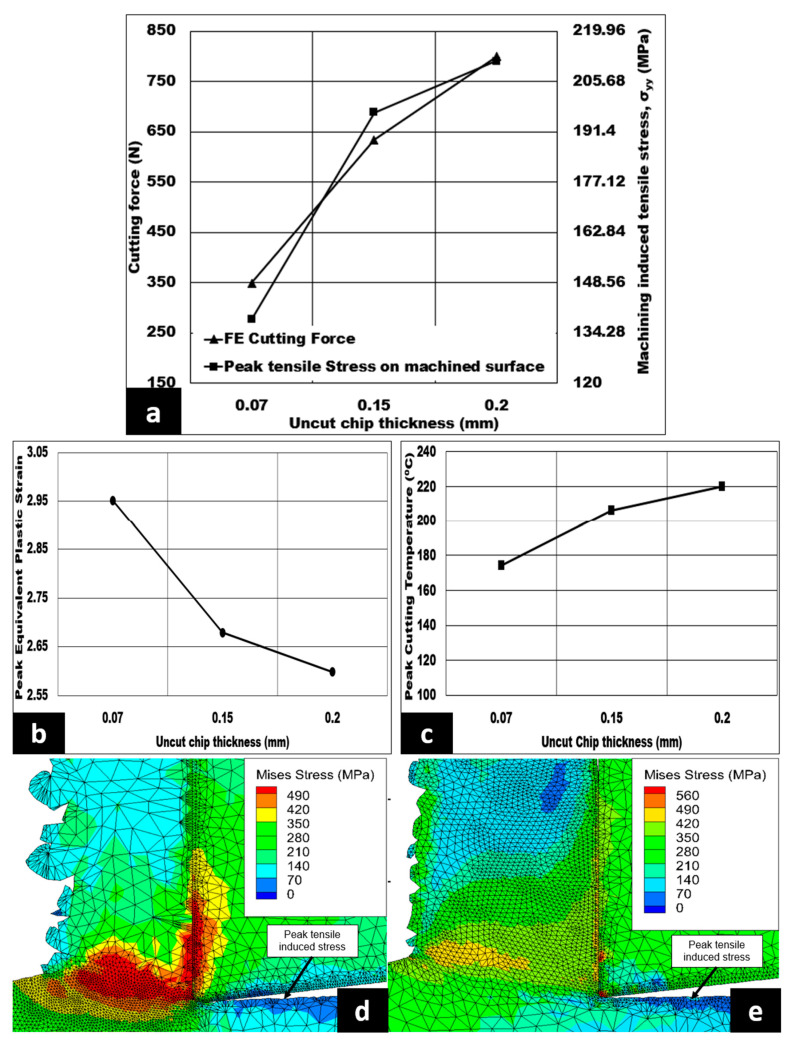
(**a**–**c**) Effect of undeformed chip thickness 7075/hollow alumina shell syntactic foams on cutting forces, machining induced stress on machined surface, peak equivalent plastic strain, and peak cutting temperature (AdvantEdge^TM^ simulation) (vol% = 10%, Vc = 50 m/min, b = 3 mm) under dry condition; Von Mises stress contour at (**d**) h = 0.07 mm and (**e**) h = 0.2 mm.

The influence of uncut chip thickness on the cutting force, cutting induced peak tensile stress (measured on the machined surface), peak equivalent plastic strain, and peak cutting temperature in a dry environment are shown in Figure 5a–c. The cutting forces are escalated in a very steep manner from 349 N to 799 N—an approximate increase of 129%—as uncut chip thickness is increased to 0.2 mm. The peak tensile induced stress on the machined surface increases by 53.11% when the uncut chip thickness increases from 0.07 mm to 0.2 mm. Moreover, the peak equivalent plastic strain drops from 2.95 to 2.6 at 0.2 mm. Additionally, peak cutting temperatures are found to increase with an increment in uncut chip thickness from 0.07 mm to 0.2 mm with an observed maximum peak cutting temperature of 220 °C (refer to Figure 5c). Figure 5d,e displays the Von Mises stress contour with the peak stress varying from 490 MPa to 560 MPa as the uncut chip thickness rises from 0.07 mm to 0.2 mm. The same figures also show the peak tensile induced stress located at the machined surface, which is highlighted by the blue contours.

As the uncut chip thickness increases, the volume of metal removed or the material removal rate (MRR) will increase. This trend is due to the involvement of a greater number of hollow aluminium oxide bubbles in pinning 7075 matrix flow. Therefore, more energy is needed to initiate plastic deformation, giving a higher cutting force which is substantiated by the Von Mises stress contour, as shown in Figure 5d,e. In addition, with the increase in cutting force, peak tensile induced stress on the machined surface is increased in magnitude, as reflected in Figure 5a. Thomas et al. [14] and Koklu and Kayhanlar [31] were able to observe trends similar to those seen in Figure 5. In the primary shear zone, this increase in number of hollow aluminium oxide bubbles will increase the chip thickness ratio, thereby raising the shear force requirement. This phenomenon supports the tendency of peak equivalent plastic strain to fall, as shown in Figure 5b. In the secondary shear zone, as noted in the simulation, the chip tool contact length (CTCL) is higher, which means that a greater number of hollow aluminium oxide bubbles could lead to a two-body and three-body abrasion against the rake face of the tool [13]. Consequently, the friction between the chip and the tool is expected to increase, which is supported by the increase in peak cutting temperature shown in Figure 5c. 

**Figure 6:**
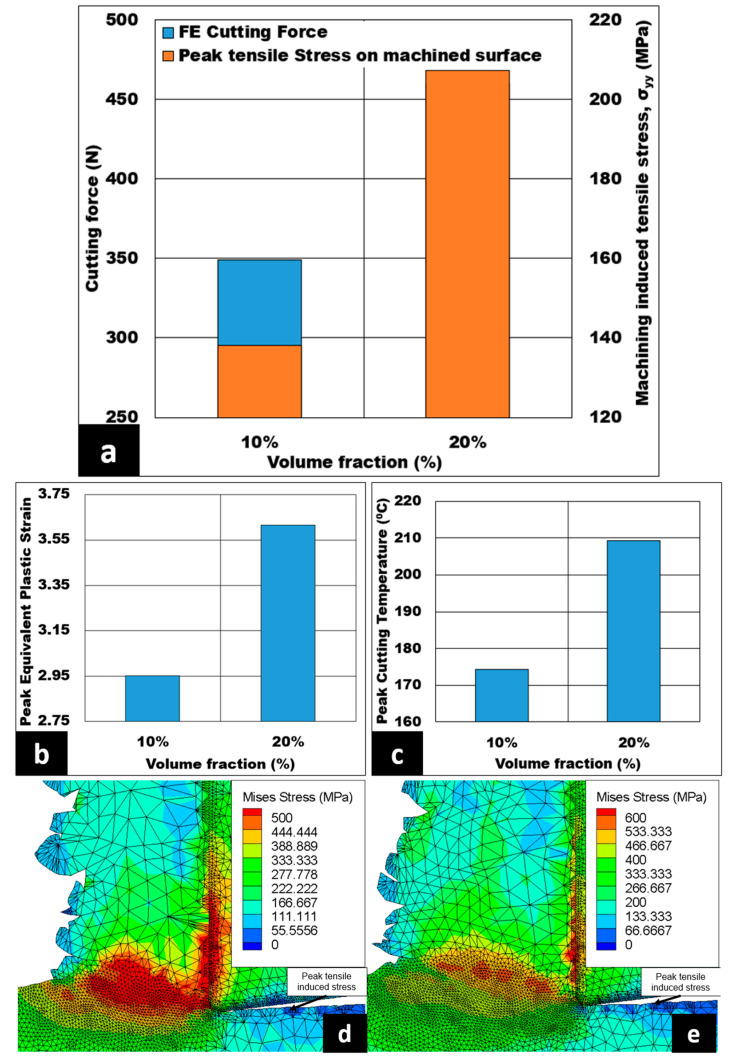
(**a**–**c**) Effect of volume fraction of alumina bubble present in 7075 on cutting forces, machining induced stress on machined surface, peak equivalent plastic strain, and peak cutting temperature (AdvantEdge^TM^ simulation) (h = 0.07 mm, Vc = 50 m/min, b = 3 mm) under dry conditions; Von Mises stress contour at (**d**) 10% and (**e**) 20%.

The effect of the volume fraction of hollow aluminium oxide bubbles on cutting force, cutting induced peak tensile stress (measured on the machined surface), peak equivalent plastic strain, and peak cutting temperature under dry cutting are presented in Figure 6a–c. The cutting forces increase steadily from 349 N to 460 N–approximately a 31.8% increase—as the volume fraction of hollow aluminum oxide bubbles increases from 10% to 20%. In addition, the cutting induced peak tensile stress on the machined surface is shown to be higher by 50% with an increase in volume fraction, as observed in Figure 6a. Moreover, the peak equivalent plastic strain increases from 2.95 to 3.62. Likewise, peak cutting temperatures increase from 174 °C to 209 °C–a nearly 22.71% increase—with an increase in volume fraction from 10% to 20%. Lastly, the peak Von Mises stress values increase from 500 to 600 MPa with an increase in volume fraction, as shown in Figure 6d,e. The same figures also exhibit the peak tensile induced stress located at the machined surface, as highlighted by the dark blue color.

With an increase in volume fraction, a greater number of hollow aluminium oxide bubbles is expected to constrain the plastic deformation of the matrix. Subsequently, this leads to a reduction in the length of the shear plane, and an increase in shear angle. As a result, the shear plane area is reduced, necessitating a higher magnitude of the shear force required to instigate plastic deformation of 7075 matrix. This in turn results in increased cutting forces (as shown in Figure 6a) causing peak tensile induced stress on the machined surface to be more tensile in nature. An increase in volume fraction also leads to a higher chip thickness ratio. Therefore, an elevation in peak equivalent plastic strain is observed, as shown in Figure 6b, which is similar to the results of the study conducted by Hameed et al. [32]. In the secondary shear zone, the CTCL is higher, with an increase in volume fraction enabling a greater number of hollow aluminium oxide bubbles to rub against the rake face of the cutting tool. Hence, the chip compression ratio decreases, resulting in higher frictional shear stress and peak cutting temperature (refer to Figure 6c).

**Figure 7:**
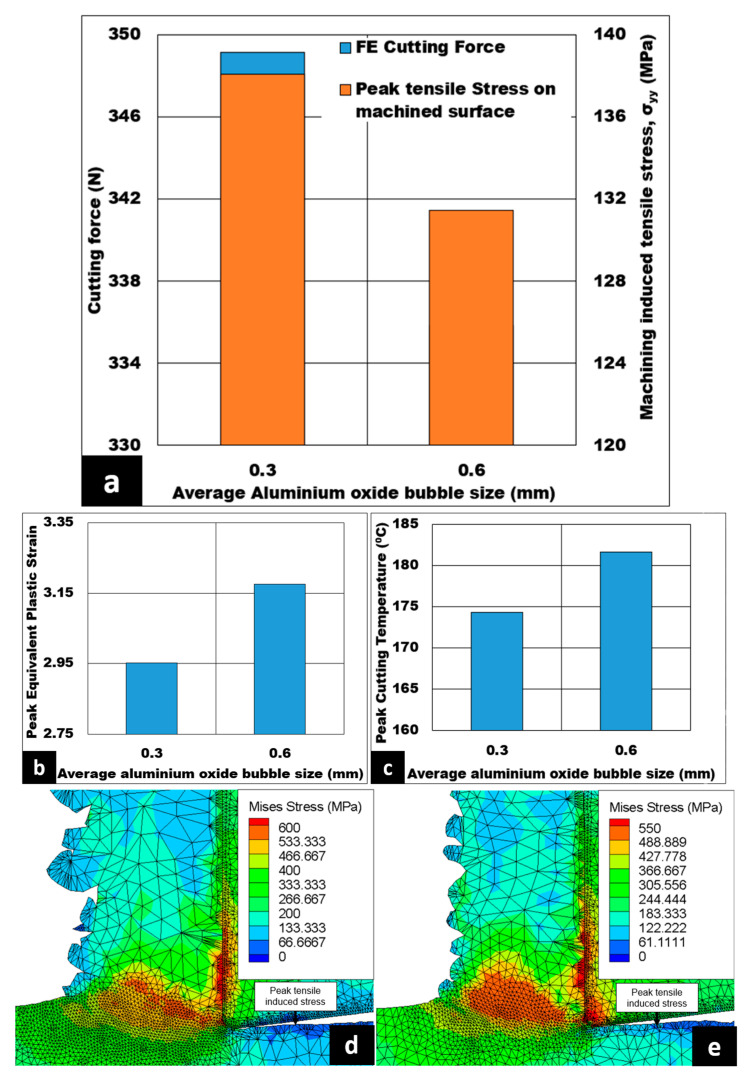
(**a**–**c**) Effect of average size of alumina bubble on cutting force, machining induced stress on machined surface, peak equivalent plastic strain, and peak cutting temperature (AdvantEdge^TM^ simulation) under dry cutting. 7075/hollow alumina shell syntactic foams (Vc = 50 m/min, h = 0.07 mm, 10% volume fraction, b = 3 mm); Von Mises stress contour at (**d**) d = 0.3 mm and (**e**) d = 0.6 mm.

Figure 7a–c show the effect of the average size of hollow aluminium oxide bubbles on the magnitude cutting forces generated, cutting induced peak tensile stress (measured on the machined surface), peak equivalent plastic strain, and peak cutting temperature under dry conditions. The cutting forces decline from 349 N to 336 N as the average size of hollow aluminium oxide bubbles becomes coarser. Moreover, cutting induced peak tensile stress declines by 4.8% when the average size of hollow aluminium oxide bubbles is increased. In addition, the peak equivalent plastic strain is observed to rise from 2.95 to 3.18, an increase of around 7.79%. Lastly, peak cutting temperature increases from 174 °C to 182 °C with coarser bubble size. Von Mises stress contours for the average size of hollow aluminium oxide bubbles are shown in Figure 7d,e. As shown by the figures, when the average size of hollow aluminium oxide bubbles becomes coarser, the peak Von Mises stress value drops from 600 MPa to 550 MPa, a roughly 8.33% reduction. In addition, the same figures also reveal the peak tensile induced stress located at the machined surface, as pointed out by the dark blue color.

If the average size is coarser at a given volume fraction of hollow aluminium oxide bubbles, it is expected to have a lesser number of hollow aluminium oxide bubbles to pin down the matrix flow, resulting in a reduced energy requirement for plastic deformation. Therefore, the cutting force is decreased as shown in Figure 7a. In the primary shear zone, the chip thickness ratio is increased, causing the shear angle to decrease and the shear plane length to increase. Subsequently, the shear plane area is enlarged, thus reducing shear force and shear stress for chip formation. This is cited as a potential reason for the fall in cutting force and a lower magnitude of machining induced tensile stress on the cut surface. An increase in chip thickness ratio could also lead to higher peak equivalent plastic strain with the foam reinforced with coarser bubbles, as shown in Figure 7b. This trend is in line with the findings of Hameed et al. [32]. As noted on the secondary shear zone in Figure 7d,e, increased CTCL could cause higher friction between the cutting tool and the chip, causing the peak cutting temperature to increase, as shown in Figure 7c. This increased peak cutting temperature softens the 7075 matrix, causing a drop in cutting force. As a verification, peak Von Mises stress values show a decrease of 8.33%, as shown in Figure 7d,e. 

### 3.2. Effect of Process Parameters on Simulated Machining Induced Stress

**Figure 8:**
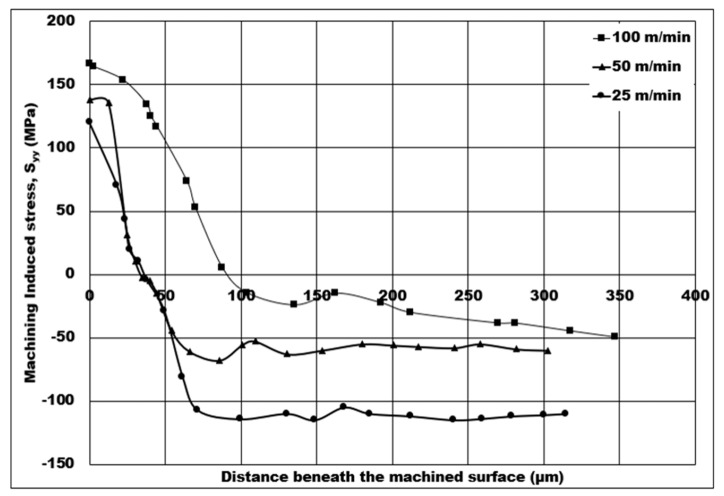
Numerical machining induced stress distribution under the influence of cutting speed (10% volume fraction, uncut chip thickness = 0.07 mm, dry cutting).

**Figure 9:**
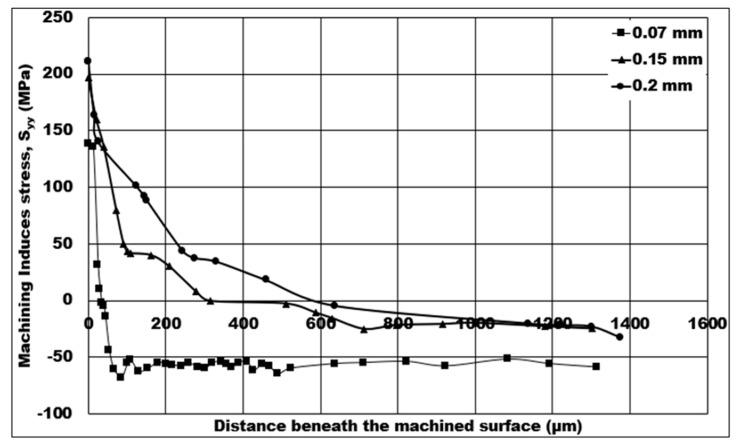
Numerical machining induced stress distribution under the influence of uncut chip thickness (10% volume fraction, cutting speed = 50 m/min, dry cutting).

**Figure 10:**
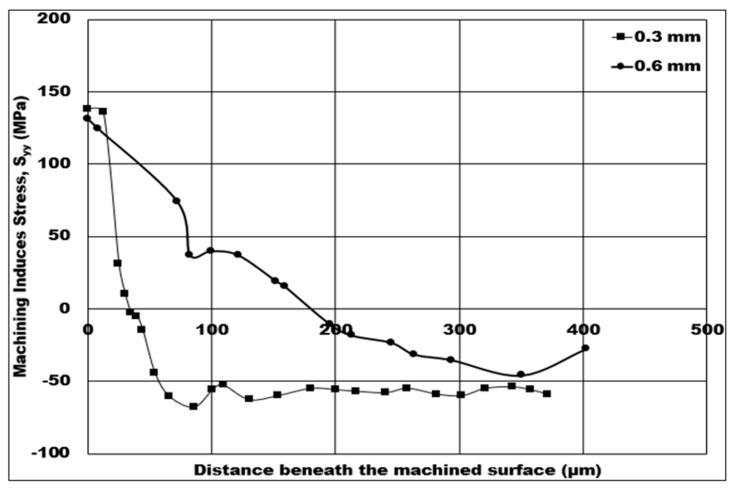
Numerical machining induced stress distribution under the influence of average-sized hollow aluminium oxide bubbles (10% volume fraction, cutting speed = 50 m/min, uncut chip thickness = 0.07 mm, dry cutting).

**Figure 11:**
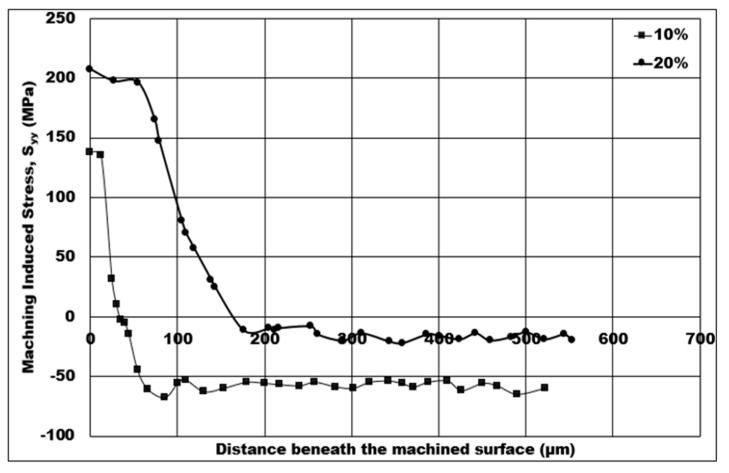
Numerical machining induced stress distribution under the influence of volume fraction of hollow aluminium oxide bubbles (10% volume fraction, cutting speed = 50 m/min, uncut chip thickness = 0.07 mm, average size = 0.3 mm, dry cutting).

**Figure 12:**
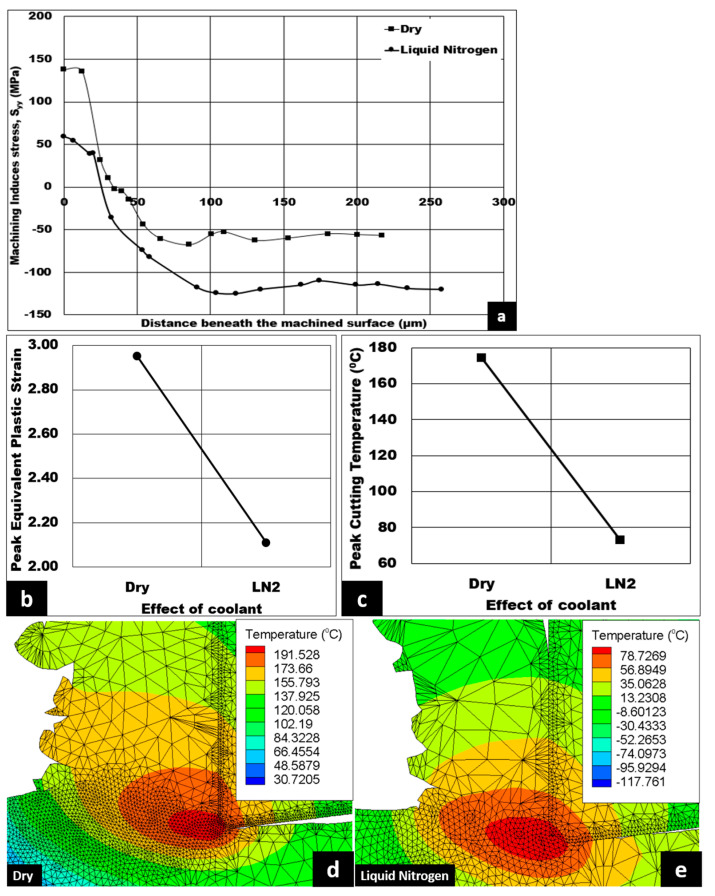
(**a**) Numerical machining induced stress distribution, and variation of (**b**) peak equivalent plastic strain and (**c**) peak cutting temperature under the influence of coolant (10% volume fraction, cutting speed = 50 m/min, uncut chip thickness = 0.07 mm, average size = 0.3 mm, volume fraction 10%); Corresponding temperature contours at (**d**) dry and (**e**) liquid nitrogen conditions respectively.

Mechanical stresses induced on the machined surface and sub-surface by the machining process can be detrimental to the component’s fatigue life in service. Therefore, it is vital to investigate and analyze the behavior of machining induced stress. In this study, the influence of machining conditions on the simulated machining induced stress is estimated using a 2D FE simulation, and analysis of the stress distribution is also conducted. The fatigue life of components will rely heavily on the depth of machining induced tensile stress. Because certain machining conditions create non-favorable tensile induced machining stress, one of the aims of this paper is to discover the influence of machining conditions on the depth of tensile induced stress distribution. In Figure 8, Figure 9, Figure 10, Figure 11 and Figure 12, tensile stress is initially distributed on below the machined surface, and, after a certain distance, distribution is later changed to compressive stress.

The influence of cutting speed on the numerical machining induced stress during orthogonal machining of 7075 syntactic foam is shown in Figure 8. The cutting induced peak tensile stress increases from 120 MPa to 166.491 MPa, an increase of approximately 38.74%, when the cutting speed is raised from 25 m/min to 100 m/min. Moreover, the depth of cutting induced tensile stress distribution increases from 35 μm to 90 μm, which amounts to a 157% increase, as the cutting speed is increased from 25 m/min to 100 m/min. The peak cutting induced compressive stress has a maximum value of 110 MPa at 25 m/min, which is further lowered to a minimum value of 50 MPa as the cutting speed increases to 100 m/min—a massive drop of 54.54% in compressive induced stress on the machined surface.

As the cutting speed is increased, higher peak cutting temperatures are experienced by the foam in the primary shear zone, as observed in Figure 4f,g. This causes the length of the shear plane to increase, leading to a higher shear plane area at higher peak cutting temperatures resulting in a drop in shear forces. In other words, a high amount of heat generation is created due to the increased peak cutting temperature, resulting in reduced average shear forces and maximum shear stress for syntactic foams. Consequently, the chip compression ratio reduces causing cutting induced peak tensile stress on the machined surface to be more tensile, and a greater depth of cutting induced tensile stress distribution. The cutting-induced stress on the machined surface is the lowest (120 MPa) at 25 m/min, which makes it satisfactory for the syntactic foam components due to a lower depth of tensile induced stress distribution (77 μm). This trend is in agreement with the findings of Thomas et al. [14].

Figure 9 shows the control of uncut chip thickness on the numerical machining induced stress distribution. The cutting induced peak tensile stress increases by 53.11% from 138.09 MPa to 211.431 MPa when the uncut chip thickness is raised from 0.07 mm to 0.2 mm. In addition, the depth of cutting induced tensile stress distribution increases in a sharply from 36 μm to 580 μm—a very large depth amount—as the uncut chip thickness increases from 0.07 mm to 0.2 mm. The peak cutting induced compressive stress has a minimum value of 32.793 MPa at 0.2 mm, which further increases to a maximum value of 60 MPa as the uncut chip thickness is lowered from 0.2 mm to 0.07 mm, a huge increase of 82.96% in compressive induced stress on the machined surface.

As the uncut chip thickness is higher, a greater number of shells are expected to come into contact with the machining tool’s rake face, which further leads to increased CTCL and friction. Subsequently, a surge in peak cutting temperature is observed at the secondary shear zone, which is also accompanied by a decrease in chip compression ratio. Therefore, the induced stress on the machined surface becomes more tensile in nature, and the depth of cutting induced tensile stress distribution is higher. The increase in tensile induced stress on the surface is found to be in agreement with Zou et al. [17]. One thing to note is an increased depth of tensile induced stress distribution with increasing uncut chip thickness, as a larger number of hollow aluminium oxide bubbles take part in enhancing the matrix hardening. The favorable condition to induce more compressive stress distribution is the lowest uncut chip thickness (0.07 mm) at a constant cutting speed. Thomas et al. [14] found a similar trend under the influence of uncut chip thickness.

Figure 10 displays how the variation in the average size of hollow aluminium oxide bubbles at a 10% volume fraction affects the numerical machining induced stress distribution. The cutting induced peak tensile stress drops slightly by 5.04%, from 138.09 MPa to 131.46 MPa, when the average size of hollow aluminium oxide bubbles becomes coarser from 0.3 mm to 0.6 mm. Furthermore, the depth of cutting induced tensile stress distribution changes from 36 μm to 177.445 μm—a huge depth increment—as the average size becomes coarser from 0.3 mm to 0.6 mm. The peak cutting induced compressive stress has a maximum value of 60 MPa at 0.3 mm, which further reduces to a minimum value of 46.07 MPa as the average size of hollow aluminium oxide bubbles is made coarser from 0.3 mm to 0.6 mm, which is a 23.21% decrease in compressive induced stress on the machined surface.

In the secondary shear zone, when the average size of hollow aluminium oxide bubbles becomes coarser, a lesser number of hollow aluminium oxide bubbles is expected to be in contact with the rake face of the tool. Unexpectedly, the depth of cutting induced tensile stress distribution is higher for 0.6 mm bubble size when compared to 0.3 mm bubble size. This trend may be due to the chip–tool contact area for 0.6 mm coarseness being slightly higher than that for 0.3 mm coarseness. However, a lesser number of 0.6 mm-coarseness hollow aluminium oxide bubbles leads to reduced pinning of 7075 matrix during plastic deformation. Therefore, the tensile induced stress on the machined surface becomes less tensile. In this case, the favorable condition would be a 0.3 mm average size of hollow aluminium oxide bubbles, since the depth of tensile induced stress distribution is lower (36 μm) despite having a slightly higher tensile induced stress on the machined surface.

The variation in the volume fraction of hollow aluminium oxide bubbles on the numerical machining induced stress distribution for 7075 syntactic foam is displayed in Figure 11. From that figure, a 10% volume fraction effects the minimum cutting induced tensile stress on the machined surface, and the lowest depth of cutting induced tensile stress distribution. The cutting induced peak tensile stress increases linearly by 50%, from 138.09 MPa to 207.25 MPa, when the volume fraction of hollow aluminium oxide bubbles is increased from 10% to 20%. Likewise, the depth of cutting induced tensile stress distribution changes from 36 μm to 160 μm (a huge depth increment) as the volume fraction spikes from 10% to 20%. The peak cutting induced compressive stress has a maximum value of 60 MPa at a 10% volume fraction, which later declines to a minimum value of 15 MPa—a 75% decrement of compressive induced stress on the machined surface—when the volume fraction of hollow aluminium oxide bubbles is higher.

If the volume fraction of hollow aluminium oxide bubbles is increased at a fixed average size of hollow aluminium oxide bubbles, a greater number of hollow aluminium oxide bubbles might be in contact with the rake face of the machining tool in the secondary shear zone. This means an increase in CTCL, which later leads to a surge in friction between the tool and the chip. Consequently, an increase in peak cutting temperature is expected, causing the depth of cutting induced tensile stress distribution to rise. In the primary shear zone, the length of the shear plane is reduced, and the shear angle increases. Subsequently, the shear force and the maximum shear stress increase. Therefore, the chip compression ratio decreases, causing the cutting induced tensile stress on the machined surface to be more tensile in nature with an increase in the volume fraction of hollow aluminium oxide bubbles. The favorable condition for this case is a 10% volume fraction, as the depth of cutting induced tensile stress distribution is at a minimum (36 μm) and the peak cutting induced tensile stress is the lowest (138.09 MPa).

Lastly, studies involving the impact of coolant on machining induced stress distribution, peak equivalent plastic strain, peak cutting temperature, and the temperature contour are displayed in Figure 12a–e. Using cryogenic coolant is known to offer better lubrication and increased effectiveness compared to wet lubrication [33]. As shown in Figure 12a, cryogenic coolant has the highest cutting induced tensile stress on the machined surface and the lowest depth of cutting induced tensile stress distribution. The cutting induced peak tensile stress drops significantly, from 138.09 MPa to 59.202 MPa (which is approximately 57.12%), when the machining condition is switched from dry to cryogenic. The depth of cutting induced tensile stress distribution decreases from 36 μm to 30 μm when cryogenic coolant is used. Furthermore, the peak cutting induced compressive stress has a maximum value of 120 MPa at cryogenic coolant, which later declines to a minimum value of 60 MPa under dry machining conditions. In addition, the introduction of liquid nitrogen results in a 28.47% drop in peak equivalent plastic strain, as shown in Figure 12b. Lastly, liquid nitrogen coolant also causes a significant drop of 58.04% in peak cutting temperature from 174 °C to 73 °C. The decrease in peak cutting temperature is supported by the temperature contours shown in Figure 12d,e, with a minimum peak temperature value of 78.7269 °C observed for liquid nitrogen coolant.

In the secondary shear zone, liquid nitrogen coolant causes the CTCL to reduce, which causes the friction between the cutting tool and the chip to drop. Consequently, peak cutting temperatures reduce, allowing the syntactic foam to be less ductile or more brittle in nature. This leads the depth of cutting induced tensile stress distribution to decrease. A reduction in peak cutting temperatures causes less thermal softening on the 7075 matrix. Therefore, the peak tensile stress on the machined surface becomes more compressive in nature. In the secondary shear zone, the observed CTCL reduces when liquid nitrogen coolant is used. This trend results in reduced secondary friction shear stress as the liquid nitrogen coolant reduces friction between machining tool and chip [34]. Therefore, the peak cutting temperature is significantly reduced under liquid nitrogen (look at Figure 12c). This trend can also be observed in the decrease in peak temperature value shown in Figure 12d,e. A reduction in peak cutting temperature might make the foam behave in a less ductile or more brittle manner, resulting in a decrease in peak equivalent plastic strain, as shown in Figure 12b. Liquid nitrogen coolant provides the most favorable condition as the depth of tensile stress distribution is lower and the peak tensile induced stress is compressive in nature. This means that the hardness value could be increased, as observed by Khaled et al., when drilling GLARE laminates and S2/FM94 glass fibre under cryogenic coolant [35,36].

## 4. Conclusions

This paper developed a numerical simulation model for 7075 syntactic foams for predicting machining induced stress using AdvantEdge^TM^ FE software. The main points to be noted from this study are as follows:

- An increase in cutting speed from 25 m/min to 100 m/min caused the machining induced surface to be more tensile in nature at higher cutting speeds up to a depth of 90 microns because of thermal softening of the matrix (as shown through peak cutting temperature contours).
- Orthogonal machining at a higher uncut chip thickness raised the cutting forces by 129%, with a corresponding increase of up to 600 microns in the depth of machining induced tensile stress beneath the cutting surface.
- The depth of machining induced tensile stress was increased by 350% for the syntactic foams reinforced with a coarser size of hollow aluminium oxide bubbles.
- Compared to dry cutting, liquid nitrogen coolant resulted in a significant (28.57%) reduction in the depth of machining induced tensile stress distribution.
- A favorable machined stress distribution was found at a cutting speed of 25 m/min, an uncut chip thickness of 0.07 mm, a 10% volume fraction, and a 0.3 mm average size of hollow aluminium oxide bubbles using liquid nitrogen coolant.
- The main novelty behind this software is the assumption of material foam as a homogeneous material model in order to simplify the material model as a complex heterogeneous material system for simulations; this enabled the production of FE results with reasonable accuracy. With this FE model developed using the AdvantEdge^TM^ software, it is possible to generate machining induced stress distribution from the contour, reducing effort in comparison to experimental work.
- Future studies must explore the addition of hollow particles to the matrix in the FE model, as well as techniques which can combine virtual reality technology with machining simulations thereby reducing unnecessary work for the technician.

## Figures and Tables

**Table 1:** Chemical composition of 7075 syntactic foam (supplier data).

Chemical Composition of 7075 Matrix in Weight (%)							
Al	Si	Fe	Cu	Mn	Mg	Cr	Zn
89.72	0.08	0.24	1.5	0.06	2.4	0.2	5.8
Chemical Composition of the alumina bubble in weight (%)							
Al_2_O_3_		Fe_2_O_3_	CaO		SiO_2_	Na_2_O	
99.7		0.003	0.01		0.025	0.26	

**Table 2:** Physical and mechanical properties of 7075 matrix and alumina bubble [23,24,25].

Alumina Bubble					
Physical Properties					
Bulk Density (kg/m^3^)	Average Porosity (%)	Average Wall Thickness (μm)	Average Bubble Size (mm)	Bubble vol%	Thermal Conductivity (W m^−1^K^−1^)
1800	83	0.04–0.08	0.3–0.6	10%, 20%	1.5
Mechanical Properties					
Crush strength (MPa)			Poisson’s ratio		
120 ± 10			0.231		
7075 Matrix					
Physical Properties					
Density (kg/m^3^)		Thermal conductivity (W m^−1^K^−1^)		Specific heat (J kg^−1^K^−1^)	
2810		143		930	
Mechanical Properties					
Poisson ratio		Compressive strength (MPa)	Yield strength (MPa)	Young’s modulus (GPa)	
0.33		330	170	71.7	

**Table 3:** Properties of the cutting tool [26,27].

Thermal Conductivity (W m^−1^K^−1^)	Coefficient of Thermal Expansion (1/K)	Young’s Modulus (GPa)	Poisson’s Ratio	Density (kg/m^3^)	Specific Heat (J kg^−1^K^−1^)
110	5.5 × 10^−6^	700	0.31	15,600	39.8

**Table 4:** Machining factors for 7075 based syntactic foams.

Matrix	7075
Reinforcement Particle	Alumina Bubble
Tool	Carbide coated inserts from Kennametal
Rake angle and clearance angle	0° and 7°
Cutting speed (m/min)	25, 50, 100
Undeformed Chip Thickness (mm)	0.07, 0.15, 0.2
Volume fraction of hollow microsphere	10%, 20%
Average size of hollow microsphere (mm)	0.1–0.5 mm, 0.5–1 mm
Width of cut (mm)	3
Lubrication	Dry, Liquid Nitrogen

## Data Availability

The data presented in this study are available on request from corresponding author.
